# The Relation between Handedness Indices and Reproductive Success in a Non-Industrial Society

**DOI:** 10.1371/journal.pone.0063114

**Published:** 2013-05-21

**Authors:** Sara M. Schaafsma, Reint H. Geuze, Jessica M. Lust, Wulf Schiefenhövel, Ton G. G. Groothuis

**Affiliations:** 1 Behavioural Biology, Centre for Behaviour and Neuroscience, University of Groningen, Groningen, The Netherlands; 2 Clinical and Developmental Neuropsychology, University of Groningen, Groningen, The Netherlands; 3 Max-Planck-Institute for Ornithology, Human Ethology Group, Andechs, Germany; London School of Hygiene and Tropical Medicine, United Kingdom

## Abstract

The evolution of handedness in human populations has intrigued scientists for decades. However, whether handedness really affects Darwinian fitness is unclear and not yet studied in a non-industrial society where selection pressures on health and handedness are likely to be similar to the situation in which handedness has evolved. We measured both hand preference and asymmetry of hand skill (speed of fine motor control, measured by a pegboard task, and accuracy of throwing), as they measure different aspects of handedness. We investigated the associations between both the direction (left versus right) and strength (the degree to which a certain preference or asymmetry in skill is manifested, independent of the direction) of handedness. We analyzed to what extent these measures predict the number of offspring and self-reported illness in a non-industrial society in Papua, Indonesia. As it is known that body height and fitness are correlated, data on body height was also collected. Due to low numbers of left-handers we could not investigate the associations between direction of hand preference and measures of Darwinian fitness. We found a positive association between strength of asymmetry of hand skill (pegboard) and the number of children men sired. We also found a positive association for men between strength of hand preference and number of children who died within the first three years of life. For women we found no such effects. Our results may indicate that strength of handedness, independent of direction, has fitness implications and that the persistence of the polymorphism in handedness may be ascribed to either balancing selection on strength of asymmetry of hand skill versus strength of hand preference, or sexual antagonistic selection. No relationships between health and handedness were found, perhaps due to disease related selective disappearance of subjects with a specific handedness.

## Introduction

Lateralization, the asymmetric distribution of function over the cerebral hemispheres, is a widely spread phenomenon throughout the animal kingdom [1], [2]. Human handedness is one of the most pronounced lateralized behavioral traits resulting from this cerebral asymmetry. Despite ample scope for environmental factors affecting the development of handedness [3], heritability of handedness is substantial, varying between 0.23 and 0.66 with around 25% of the variance in hand preference explained by additive genetic effects [4]. Because handedness is a heritable trait, it may be subject to natural selection. Handedness can be investigated in terms of direction (left vs. right-handedness) and in terms of strength. Both can be applied to the subject’s preference for hand use (which hand is preferably used for a certain task) and the subject’s skill in using either hand (is the task performed better with the right or left hand). Most studies have focused on direction of hand preference because it is easily determined, often by self report, but the results in relation to fitness estimates are ambiguous. Non-right hand preference (in the literature sometimes classified as left- and/or mixed-handedness) has been associated with possible fitness costs such as extremely low birth weights, delayed maturation, birth stress (for references see [5], [6] (but see [7])) and auto-immune diseases [8], [9]. Besides these disadvantages, some advantages for this group have also been found. Non-right-handers are more prevalent among the top echelons of interactive sport competitors suggesting that non-right-handers have increased chances of winning these interactions compared with right-handers and this may translate to winning aggressive interactions too [1]. Furthermore, they are reported to be more common among (instrumental) musicians [10]–[14]), artists [14], [15], and people of higher socio-economic status (higher income and position in companies) [16] possibly leading to benefits in sexual selection [17], [18]. In addition, hand preference is often found to be associated with cognitive performance, although the results are ambiguous (reviewed in [16]). Whether these associations translate into differential fitness in terms of reproductive success is unclear. McManus and Bryden [19] reviewed the genetics of handedness and they found that parents of whom one was right-handed and one left-handed reported to have fewer offspring than two right-handed parents and more than two left-handed parents (table 6, [19]). Unfortunately, that observation could not be statistically tested. Faurie et al [20] investigated the association between handedness and reproductive success in French adults. They found an interaction between direction of handedness and income, such that left-handed men with low income have the lowest number, and left-handed men with high income had the highest number of grandchildren relative to right-handed men.

Strength, independent of direction, of handedness is harder to determine and studies investigating the costs and benefits of strength of handedness are scarce. Nettle [21] showed that the results of most studies that find right-handers to outperform left-handers (or non-right-handers) in cognitive tasks are actually confounded by the fact that right-handers are generally more strongly lateralized than left-handers, and that it is strength of lateralization, independent of direction, that is associated with cognitive ability. This highlights that attention should be paid to both direction and strength of handedness when investigating its advantages and disadvantages.

Asymmetry in skill, or performance, between the hands is relatively independent of hand preference as they do not show a strong correlation [22]–[25]. In contrast to hand preference, the relationship between direction or strength of hand skill and reproductive success has not yet been investigated. Although no evidence is currently available concerning the heritability of asymmetry of hand skill it can as well be asymmetry of hand skill as hand preference that is under natural selection pressures.

In this paper we intended to investigate the association between both direction and strength of handedness and reproductive success, with handedness measured both in terms of preference and skill. This study was performed in a non-industrial society, as investigating the relationship between handedness and Darwinian fitness aspects in Western societies entail possible limitations as these societies may be no longer under the selection pressures in which handedness has evolved. As we found only few individuals with a left hand preference the association between direction of hand preference and reproductive success could not be investigated. Reproductive success of subjects was estimated based on the number of their children born, alive and deceased in the first three years of life. We also investigated whether the associations found are mediated through serious health problems. The study was carried out in a non-industrial society in the highlands of Papua, Indonesia. We measured hand preference on 10 ecologically relevant tasks and asymmetry of hand skill by means of a pegboard task that measures speed of fine motor control, and a ball throwing task that measures the accuracy of eye-hand coordination over longer distance.

## Methods

### Subjects

The Eipo people inhabit an area of about 150 km^2^ near the Eipomek river at approximately 4°25′–4°27′ S, 140°00′–140°05′ E in the highlands of the Indonesian province of Papua, formerly known as Irian Jaya (New Guinea). The Eipo are horticulturists whose staple food consists of sweet potatoes and vegetables, complemented by the products of hunting, gathering and pig raising [[26], [27] and personal observations, 2009]. Because of the remoteness and inaccessibility of the area the Eipo valley is located in, it has until recently been isolated from the outside world. The first brief contact with Europeans was in 1959 during the expedition led by Gaisseau [28], and more frequent contacts only began in 1974 when the interdisciplinary German Research Team began fieldwork there [27]. Nowadays, the area is still accessible by foot or light aircraft only. Western health care was absent until 2005 when a health centre was built which now offers basic health care.

The sample comprised 373 subjects (197 women and 176 men), and is a subsample of a larger dataset collected by the first two authors during a three months field survey executed in the three major villages of the valley in 2009–2010. Criteria for inclusion in this study was the minimum age, in this population, for women to give birth to their first born (19 years of age) and for men to sire their first born (20 years of age).

Our study was approved by the ethical committee of the Psychology Department of the University of Groningen. All subjects were recruited on a voluntary basis. As most subjects were illiterate they received verbal information about the survey in their local language (*Eipo yupe*) and those who asked for additional information before, during or after their participation were further informed. The subjects were informed about the possibility to abort their participation at any point in time. Those subjects that did not explicitly give their consent or did not want to participate were excluded from the study and not recorded, and those who did were recorded. This procedure was approved by the ethical committee.

### Interviews: Health Status and Reproduction

Each subject participated in a 30 minute interview lead by one of the two first authors who was assisted by one of three local assistants who translated between the Indonesian and the local language. After establishing a subject’s name, we estimated the age of the subject as they do not record dates of births. Estimation of age was done by means of a time scale of the community’s major events of which exact dates were known from documented records [41] and the replies of subjects to questions whether they were old enough to recollect these events. Also the age of their first and last born was estimated and sometimes of other children too. When not all children’s ages were estimated, the ages were estimated by interpolation based on the ages of their siblings.

Number of children born and number of children deceased were recorded, as was the age of the child when it died. As in many cases both parents volunteered and/or information was obtained from siblings, the data could be and were cross-validated. The subjects were also questioned whether they themselves ever experienced severe (life threatening) illness or injury. After the interview and the measurements of handedness (see below), the height of the subject was measured using a straight bough on which a scale was drawn, since height has been shown to correlate with reproductive success (e.g. [29]).

### Handedness Preference Measures

Hand preference was observed during a series of tests consisting of 10 ecological relevant actions the subjects completed using the tools provided, and comprised both fine and gross motor skills. The tasks included: (1): one punch at a bag held up by an observer (mimicking giving someone a punch during a fight); (2): sharpening a wooden stick with a knife (hand used to handle knife was recorded); (3): hammering a wooden stick into the ground with a stone (hand used to handle stone was recorded); (4): machete use (imitating cutting vegetation); (5): throwing a small stone far away; (6): picking up a nut and putting it in the mouth; (7): picking up a small bead and handing it over to the observer; (8): drawing a circle on the ground with a wooden stick; (9): chasing away an imagined fly located on the subject’s nose and (10): crushing a small stone with a big stone. The tasks comprised of both unimanual (nr. 1, 4, 5, 6, 7, 8, 9 and 10) and bimanual items (nr. 2 and 3). The tools were bimanually placed precisely in front of the subject in such a way that no cue for left- or right-hand use was given by a bias in the placement of tools. A score of -1 per task was given when performed with the left hand and +1 when performed with the right hand. The scores of the ten tests were then summed up and divided by the number of tests and ranged from -1 (extreme left handed) to 1 (extreme right handed). If a person did not execute the task as instructed (e.g. by using two hands in the unimanual tasks) that person was discarded in the analysis concerning hand preference. The tests were validated in a student population that executed the tasks we designed for the Papuan population, completed a questionnaire widely used in Western populations, and performed action tasks designed for a Western society ([30], see also [31], [32]). High correlations between these measurements showed that the test series we designed for the Papuan population indicates handedness reliably in our student population [33].

In analyses concerning *direction* of hand preference we used a score of <0 to considered a person left-handed. In analyses concerning *strength* of hand preference we used the absolute value of the total score on the handedness test (see section Analyses for more details). Due to the very low sample size of individuals with a left hand preference (3 women and 7 men) direction of hand preference was omitted from the analyses (see below and [33] for discussion about the low level of left-handedness in this population) and only strength of hand preference was investigated.

### Asymmetry in Hand Skill Measures

Two measures of the asymmetry in hand skill were conducted.

#### Asymmetry of fine motor control as measured with a pegboard task

To measure the speed of fine motor control of both hands a pegboard task was used. This task was based on the apparatus designed by Annett ([34], p. 208) and has been validated and shown to be a reliable tool to measure hand skill asymmetry [35], [36]. The pegboard was constructed of hardwood (40 cm×21 cm×2.5 cm) with 2 parallel rows of 10 holes (rows 17 cm apart; holes spaced 3.75 cm apart each, Ø 0.5 cm, depth 1.2 cm). The pegboard was laid on a 50×40 cm plywood plate and placed on the lap of the subject who was sitting on a low flat rock. Ten steel pegs (Ø 0.45 cm, length 3 cm) were placed in the row of holes nearest to the subject. The subject fixed the apparatus in position with one hand, whereas the other performed the test (left and right hands in alternating order between the subjects). Subjects were instructed to move the pegs one by one to the equivalent hole at the other side of the pegboard, starting from the side of the hand used in the task. Each trial started with a practice trial moving 3 pegs forth and back. Next, the subject was instructed to move all pegs as fast as possible. The time to complete the task was recorded with a stopwatch. Subsequently the board was rotated and the procedure was repeated for the other hand. If a peg was dropped the trial was repeated for both hands. The time it took to move all pegs with the right hand was subtracted from the time it took to move all pegs with the left hand (L–R). A positive score indicates quicker peg moving ability with the right hand than with the left hand. For statistical analyses, direction of hand skill asymmetry was defined as 0 for individuals who had a score <0 (faster with the left hand) and 1 for individuals with a score >0 (faster with the right hand). Strength of hand skill asymmetry was defined as the absolute value of the left hand score minus the right hand score (|L–R|).

#### Asymmetry of accuracy of the eye-hand coordination as measured with a ball throwing task

While the pegboard is a test for asymmetry in eye-hand coordination in fine motor control over a short distance, the ball throwing task tests the asymmetry in eye-hand coordination over a longer distance and the hand preference of throwing is known to be strongly lateralized [37]. In order to test the asymmetry of skill in this task, in addition to hand preference, we also measured the accuracy of the throws for each hand. Subjects were standing 2.7 metres from a target (2 metres for a few elderly people lacking force) that was placed at eye height (approximately 1.5 metres). The target was a black cloth with circular bands printed on it. The white circle in the middle (Ø 7 cm) was surrounded by four circular bands (width 2.8 cm each and alternating black or white). The subjects were instructed to throw a tennis ball at the middle of the target and, if hit, were rewarded with fifty points. Each adjacent circle was rewarded by 10 points less compared to the band closer to the bull’s eye. If subjects threw the ball outside of the four circular bands, but closer than 2.8 cm from the outer band (as was estimated by one of the first two authors) no score was awarded. If the ball landed outside this ‘zero band’ the scores −10 to −50 were rewarded depending on the landing position away from the target area. In the rare event that the landing position was further away than the −50 band (i.e. more than 34 cm from the zero band) −50 was recorded. Subjects performed 5 trials with each hand (start with left or right hand was alternated between individuals). The score of each throw was observed from a position just behind and slightly aside the participant and noted on a scale of −50 to +50. The scores for each hand were then summed up and the total score of the left hand was subtracted from the total score of the left hand (R–L). A positive score thus indicates better ball throwing ability with the right hand than with the left hand. This calculation is the reverse of the calculation used in the analysis in the pegboard task (where L–R was used; see above) because in this way in both calculations a positive score indicates a better performance with the right hand.

For statistical analyses, direction of hand skill asymmetry was defined as 0 for individuals who had a score <0 (more accurate with the left hand) and 1 for individuals with a score >0 (more accurate with the right hand). Strength of hand skill asymmetry was defined as the absolute value of the right hand score minus the left hand score (|R–L|).

### Analyses

#### The dataset

**Figure 1:**
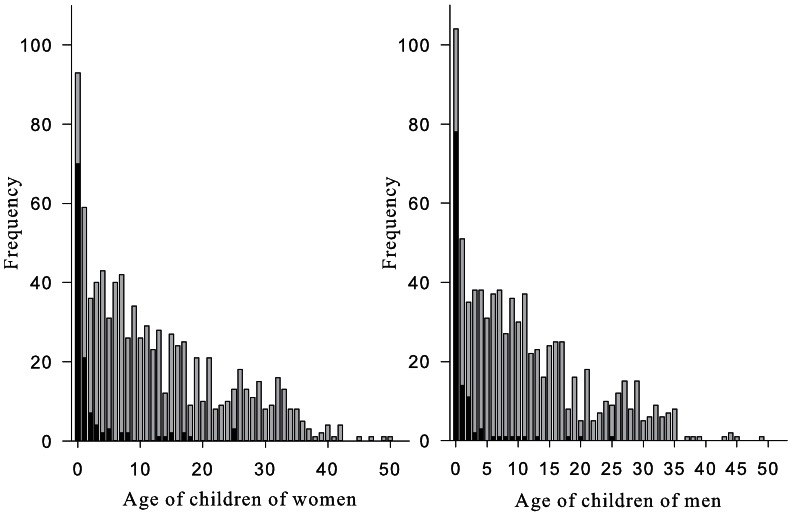
Histograms of number of children alive at the time of research (grey) and deceased (black) per age group of mothers (left panel) and fathers (right panel).

The 197 women who participated in this study gave birth to 885 children of which 124 died (98 before their 3rd birthday) and 176 men sired 815 children of which 118 died (103 before their 3rd birthday) (Figure 1, Table 1).

**Table 1:** Number of children born, died within 3 years of life and still alive at the time of the study.

	to women	to men
Children born	885	815
Nr. of children died <3 yrs	98	118
Nr. of children alive	762	696

#### Number of children born and alive

To model the relationship between the number of offspring born or surviving and handedness scores of the parents we corrected for parental age and age squared as the relationship between parental age and these two dependent variables was quadratic. We also included parent’s height in the analyses as height is shown to be related to number of offspring (e.g. [29]). The effect of handedness measures on the number of offspring, total born or still alive, was modeled with a negative binomial regression model with a logarithmic link [38] in the software program SPSS 16.0.

Due to the unexpected low sample size of individuals with a left hand preference (n = 10) (see below and [33]) we could not investigate the association between direction of hand preference and the number of offspring.

To analyze the predictive value of the strength of parental hand preference on the number of children born and still alive at the moment of the study we used the following model: Number of offspring = age+age^2^+ height+strength of hand preference. Strength of hand preference was defined as the absolute value of the total score on the handedness test battery for preference.

The data concerning hand skill asymmetry (ball and pegboard tasks) were modeled: Number of offspring = age+age^2^+ height+(Score L+R)+direction of hand skill asymmetry (left or right)+strength of hand skill asymmetry+interaction between direction and strength of hand skill asymmetry. Following Nettle [21] the left plus right hand score (L+R) score was incorporated to control for the overall performance in the task independent from the asymmetry. As direction is thus a binary variable (0 or 1; left or right) and strength of lateralization is a continuous variable (|L–R| in the pegboard task or |R–L| in ball throwing task), the latter variable is more informative than the first. However, this distinction allows us to investigate both strength and direction of the variable separately. The interaction effect of strength times direction of handedness was included in the model to be able to differentiate between possible differential effects of strength of handedness between left- and right-handers on number of children. In order to interpret the main effects of direction and strength of handedness on the number of children the models were rerun without the interaction effect.

The analyses were performed for men and women separately because the response variables of men and women were not independent as in some cases both parents of the same children were included in this study, while the effects of handedness may be sex-dependent. The fit of the models with the data was satisfactory, no overdispersion was present (scaled deviance/df <1.5 in all models).

#### Mortality of children in the first three years of life

In the first three years of life mortality is very high in this population (Figure 1). Therefore we investigated whether parental handedness influenced the survival chances in the first three years of life. In this analysis we had to consider the hierarchical structure of the data since the survival of children from the same parent may be not independent. We used two separate (fathers and mothers) two-level hierarchical logistic regression models (level 1 estimated variation in mortality at the child level, level 2 estimated variation at the parent level) using RIGLS (restricted iterative generalized least squares) estimation (MLWin 2.02 [39]) for binomial models as survival analysis. Again, the models were corrected for parental age, height, and, when asymmetry of hand skill was addressed, total score on the tasks (L+R).

#### Self-reported illness

As we found a sex-specific effect of strength of handedness on reproductive success (see section Reproductive Success) we investigated whether this effect could be mediated by the individual’s chance of ever having been exposed to severe illness. Forty one of the 197 women and 57 of the 176 men reported to have ever experienced a life threatening illness or injury. We used a logistic regression model for binary response variables (1: suffered from almost lethal illness or injury; 0: did not suffer from almost lethal illness or injury) in the software program SPSS 16.0. We included sex and controlled for the age of the subject, as older individuals would have had more time to have suffered from illnesses or injuries, and for total score on the tasks (L+R) when asymmetry of hand skill was addressed. The fit of the models with the data was satisfactory as no overdispersion was present (scaled deviance/df <1.5 in all models). These analyses were conducted on both sexes together. As no significant effect of sex was present, the models were not rerun per sex.

## Results

Table 2 gives an overview of the descriptives of the age, number of children, serious health threats, and asymmetry measures of handedness of men and women. The asymmetry of hand skill as measured with the pegboard task (L–R) and with the ball throwing task (R–L) correlated only very weakly with hand preference (Spearman’s r = 0.066 and r = 0.163, respectively).

**Table 2:** Sample size, mean, standard deviation (std. dev.), minimum and maximum of variables used in the statistical models following a normal distribution and median, variance, minimum and maximum of the variables not following a normal distribution.

		N	Mean	Std. Dev.	Minimum	Maximum	Median	Variance
Height (cm)	women	196	143.9	5.6	128	157		
	men	176	150.7	5.8	133	166		
Asymmetry of hand skill	women	192	.98	1.7	−5.3	5.7		
(Peg moving time L–R)	men	175	1.07	1.7	−3.6	5.7		
Asymmetry of fine motor control	women	196	53.2	81.9	−145	295		
(Ball throwing score R–L)	men	176	75.0	75.0	−115	275		
Total score on ball throwing task	women	196	642.5	93.6	400	860		
(Ball throwing score R+L)	men	176	686.1	86.6	405	880		
Total score on pegboard task	women	192	31.9	5.1	23.8	53.1		
(Peg moving time L+R)	men	175	32.7	5.0	24.4	50.4		
Age of subject	women	197			19	68	33.0	172.0
	men	176			21	69	44.5	133.3
Nr. of children per parent born	women	197			0	11	5	7.45
	men	176			0	11	5	7.85
Nr. of children per parent alive	women	197			0	9	4	5.45
	men				0	8	4	5.84
Self-reported illness*	women	197			0	1	0	.17
	men	176			0	1	0	.22
Direction of hand preference	women	195			−1	1	1	.08
(left(−1) versus right(1))	men	174			−1	1	1	.14
Strength of hand preference	women	195			.0	1.0	1	.02
(Absolute value of hand preference)	men	174			.2	1.0	1	.01
Direction of hand skill in pegboard task	women	192			0	1	1	.19
(left(0) versus right(1))	men	175			0	1	1	.21
Strength of hand skill in pegboard task	women	193			0	6	1.3	1.36
(Absolute value of pegboard score L–R)	men	175			0	6	1.2	1.70
Direction of hand skill in ball throwing	women	196			0	1	1	.19
(left(0) versus right(1))	men	176			0	1	1	.10
Strength of hand skill in ball throwing	women	196			0	295	75.0	3065.0
(Absolute value of ball score R–L)	men	176			0	275	72.5	4049.4

* 41 women and 57 men reported to have suffered from an almost lethal illness or injury.

### Reproductive Success

We found a significant positive association between male height and number of children alive at the time of the study (Table 3, results of the variable ‘height’ varies slightly between the models because of the slightly different number of subjects, see Table 2). This result confirms earlier studies that linked human height and reproductive success in men (eg. [29]). As mentioned in the section *Number of children born and alive*, in all models we controlled for height when investigating the relationship between handedness measures and reproductive success. However, the overall results do not change when height is left out of the models (data not shown).

**Table 3:** Regression analyses of each association between different measures of handedness and components of reproductive success.

Variable	Hand preference					Pegboard task					Ball throwing task				
	Predictor	B	SE	Wald	p	Predictor	B	SE	Wald	p	Predictor	B	SE	Wald	p
**Number of children born**															
Men	Height	1.444	0.772	3.499	0.061	Height	1.324	0.743	3.179	0.075	Height	1.489	0.772	3.721	0.054
	Strength	0.144	0.392	0.136	0.713	Strength	0.074	0.032	5.260	0.022	Strength	−0.001	0.001	0.564	0.453
						Direction	−0.050	0.091	0.304	0.581	Direction	0.017	0.137	0.016	0.901
						Direction×Strength	−0.023	0.086	0.074	0.785	Direction×Strength	0.002	0.004	0.345	0.557
Woman	Height	0.599	0.656	0.835	0.361	Height	0.518	0.673	0.592	0.442	Height	0.463	0.667	0.483	0.487
	Strength	−0.021	0.224	0.924	0.979	Strength	−0.011	0.030	0.123	0.725	Strength	0.000	0.001	0.048	0.826
						Direction	0.010	0.081	0.015	0.902	Direction	−0.060	0.078	0.592	0.442
						Direction×Strength	0.056	0.070	0.650	0.420	Direction×Strength	0.002	0.002	1.810	0.178
**Number of children alive**															
Men	Height	1.865	0.753	6.128	0.013	Height	1.685	0.741	5.171	0.023	Height	1.855	0.756	6.021	0.014
	Strength	−0.232	0.366	0.402	0.526	Strength	0.060	0.032	3.378	0.066	Strength	−0.001	0.001	0.553	0.457
						Direction	−0.072	0.090	0.639	0.424	Direction	0.028	0.135	0.042	0.838
						Direction×Strength	−0.027	0.084	0.106	0.745	Direction×Strength	0.002	0.004	0.409	0.522
Woman	Height	0.688	0.708	0.946	0.331	Height	0.640	0.726	0.777	0.378	Height	0.578	0.722	0.643	0.423
	Strength	−0.006	0.243	0.001	0.981	Strength	−0.004	0.032	0.014	0.906	Strength	0.114	<0,001	<0,001	0.736
						Direction	−0.004	0.087	0.002	0.963	Direction	0.012	0.085	0.021	0.886
						Direction×Strength	0.057	0.075	0.581	0.446	Direction×Strength	0.129	0.569	0.051	0.821
**Number of children deceased in the first three years of life**															
Men	Height	−3.740	2.238	2.793	0.095	Height	−3.635	2.289	2.521	0.112	Height	−3.648	2.254	2.620	0.106
	Strength	6.215	2.508	6.142	0.013	Strength	0.073	0.089	0.672	0.412	Strength	<0,001	0.002	0.035	0.852
						Direction	0.062	0.290	0.045	0.832	Direction	0.295	0.423	0.486	0.486
						Direction×Strength	0.264	0.299	0.776	0.378	Direction×Strength	−0.005	0.012	0.156	0.693
Women	Height	2.143	2.147	0.997	0.318	Height	2.456	2.183	1.266	0.261	Height	1.784	2.183	0.668	0.414
	Strength	−0.743	0.631	1.384	0.239	Strength	−0.126	0.098	1.636	0.201	Strength	−0.003	0.002	1.832	0.176
						Direction	0.384	0.277	1.927	0.165	Direction	−0.241	0.240	1.004	0.316
						Direction×Strength	0.400	0.384	1,083	0.298	Direction×Strength	0.002	0.006	0.181	0.671

B’s, standard errors (SE), Wald Chi square statistics and p-values are presented. Results of the interaction effects are reported from the full models whereas those of the main effects are from models without the interaction effects. The models additionally include age, age^2^, and the total score on the task (L+R) in the models concerning the pegboard or ball throwing task. For more details please refer to the text.

**Figure 2:**
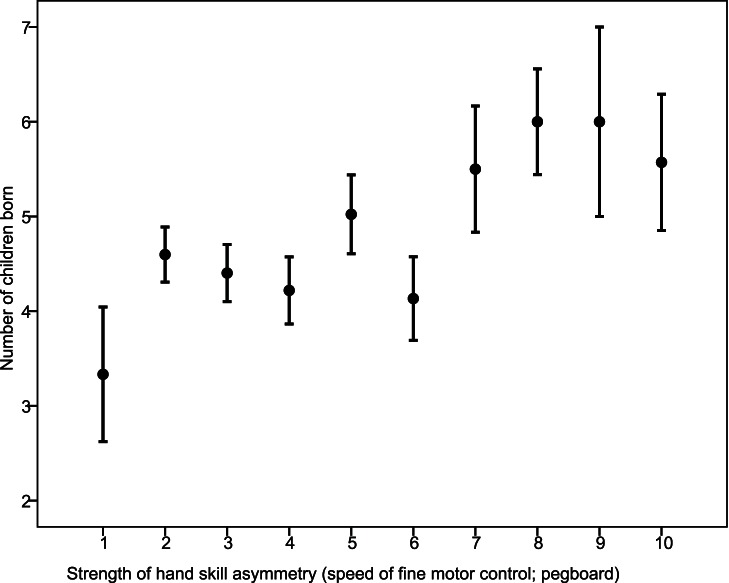
The relationship in fathers between strength of hand skill, measured with the pegboard task and for graphical purposes categorized in 10 groups of equal widths, and number of children born (mean and standard errors).

**Figure 3:**
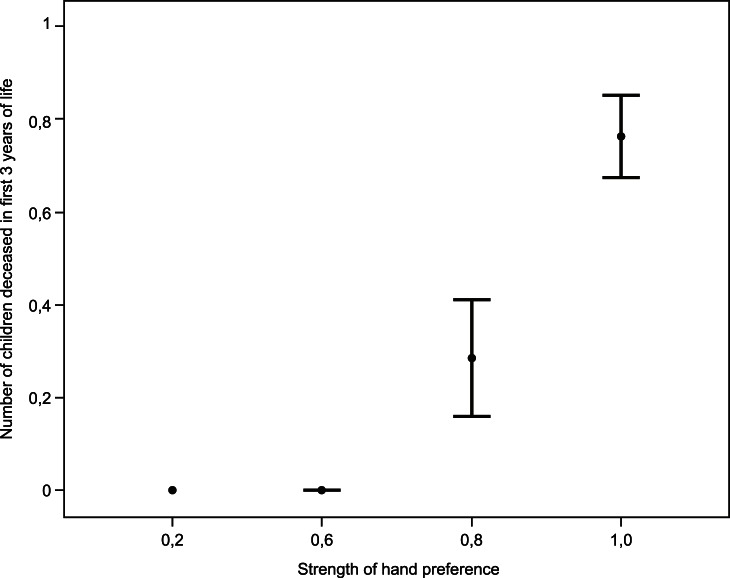
The relationship in fathers between strength of hand preference and number of children deceased in the first three years of life (mean and standard errors).

In females neither hand preference nor asymmetry of hand skill showed to be associated with any of our measures of reproductive success (Table 3). In contrast, in men several statistically significant associations between handedness and components of reproductive success were found (Table 3). A positive relationship was found between strength of fine motor control (as measured with the pegboard task) and the number of children born: men who showed a strong asymmetry in fine motor control sired more children (Figure 2). No relationship between child mortality during the first three years of life and this aspect of handedness was found in men. As to be expected on the basis of these results, strongly lateralized men, as measured with the pegboard task, showed a trend to have more children alive than weakly lateralized men, but this did not reach statistical significance (p = 0.066, Table 3). Furthermore, children from men with a strong hand preference died more often before the age of 3 (Table 3, Figure 3). However, this did not significantly affect the number of children alive. No relationships between handedness as measured with the ball throwing task and components of reproductive success were found (Table 3).

### Self-reported Illness

26.3% of the subjects reported to have suffered at least once from a severe illness during their lifetime. We found no significant associations between self-reported severe illness and handedness measures. We found a non-significant negative trend between asymmetry of accuracy of eye-hand coordination over longer distance as measured by a ball throwing task and self-reported illness, i.e. subjects showing high asymmetry of hand accuracy showed a non-significant trend (p = 0.089) to have suffered less from a severe illness (Table 4).

**Table 4:** Regression analyses of the associations between different measures of handedness and self-reported illness.

Hand preference					Pegboard task					Ball throwing task				
Predictor	B	SE	Wald	p	Predictor	B	SE	Wald	p	Predictor	B	SE	Wald	p
Strength	0.179	1.032	0.030	0.862	Direction×Strength	0.168	0.284	0.350	0.554	Direction×Strength	−0.008	0.008	1.146	0.284
Sex	0.401	0.251	2.550	0.110	Direction	0.216	0.291	0.551	0.458	Direction	−0.091	0.323	0.078	0.779
					Strength	−0.108	0.110	0.965	0.326	Strength	−0.004	0.002	2.899	0.089
					Sex	0.340	0.253	1.800	0.180	Sex	0.401	0.262	2.347	0.126

B’s, standard errors (SE), Wald Chi square statistics and p-values are presented. Results of the interaction effects are reported from the full models whereas those of the main effects are from models without the interaction effects. The models additionally include age, and total score on the task (L+R) in the models concerning the pegboard or ball throwing task. For more details please refer to the text.

## Discussion

This study presents data on the associations between direction (left versus right) and strength (independent of direction) of handedness and reproductive success in a non-industrial society. We studied both asymmetry of skill of the hands and hand preference and in concordance with the literature [22]–[25] we found that these measures of handedness are only weakly correlated. This weak correlation may be due to the different shapes of their distributions, but may also reflect different biological aspects of lateralization. Due to the latter possibility and the fact that the effect sizes of both are difficult to compare they will be discussed separately.

### Hand Preference

#### Strength of preference

We found a positive association between strength of hand preference in men and the number of children who die within the first three years of life. Although men who showed a strong hand preference thus tended to father more children who died within the first three years of life than men who showed a weak hand preference, they did not sire more offspring who were still alive during the time of research, even though there was no difference between strongly and weakly lateralized men in the number of total sired children. This discrepancy can be caused by a reduction of statistical power in the latter two analyses: Since the majority of children survived the first three years of life, the number of deceased children does only marginally affect the number of surviving children.

#### Direction of hand preference

Our sample consisted of a surprisingly low number of individuals with a left hand preference (3 women and 7 men). This finding undermines the fighting hypothesis, which states that the frequency of left-handers in a non-industrial population should be high when homicide rates are high [40]. As in the Eipo population homicide rate was very high until recently, the fighting hypothesis predicts a high percentage of left-handers in this population, a prediction that our study could not support. This finding is discussed in our previous paper and argues that possibly the low accessibility of modern health care in this population could negatively affect the frequency of left-handers as the latter is associated with diseases [33]. In addition to health care other environmental factors could also influence the frequency of left-handers. Schooling, for example, is associated with hand preference in this population (with schooled individuals having a stronger right-hand preference than non-schooled individuals) and is possibly mediated by the specific and intense training of fine motor control of one hand in school and at the same time the reduced time spent in the gardens which are situated on steep slope or going up into the forests for hunting and trapping, activities that are physically demanding and require varied use of both hands [41].

Because of the low frequency of left-handers in this population the possible effects of direction of hand preference on reproductive success could not be investigated. Thus, we can not support or oppose the observation of McManus and Bryden [19] and Faurie et al [20] concerning direction of handedness and reproductive success. However, as in general left-handers are less strongly lateralized than right-handers [e.g. [21], it may well be possible that in the studies of Faurie et al [20] and McManus and Bryden [19], who only had information on direction and not on strength of handedness, it was actually strength, as in our study, and not direction of handedness that underlay their observed association between handedness and reproductive success.

### Asymmetry of Hand Skill

In contrast to associations concerning the direction of hand preference, associations concerning the direction of hand skill could be reliably investigated as sample sizes of both groups (performing better with left hand or performing better with right hand) were substantial. We found no associations between direction of lateralization in skill and reproductive success, suggesting that direction of lateralization in skill does not affect Darwinian fitness. As was the case for hand preference, we did find a significant association concerning the strength of lateralization. Strongly lateralized men, as measured in the pegboard task, sired more children than weakly lateralized men. We are aware of the problem of multiple testing and a Bonferroni correction would annul the significant result. Nevertheless, very weak pressures from natural selection on traits (as shown by low effect sizes) can still be of great importance for the persistence of traits. Thus, even though we can not draw any firm conclusions, our finding may suggest that strength of lateralization in skill is positively associated with reproductive success. Since this was found for the pegboard and not for the ball throwing task the results suggests that lateralization of fine motor skills is more strongly under natural selection.

At this stage we can only speculate about how laterality indices are translated into fitness estimates. There is evidence that cognitive ability increases with increasing strength of asymmetry of hand skill in either direction [21]. This may explain why a strong asymmetry of hand skill might lead to higher number of children sired, either via genetic effects, or to higher quality of mothers that are assortatively paired with higher quality males. Additionally, stronger lateralization of fine motor skill may be related to better performance in tool use and hunting skills, but this remains to be tested.

### Persistence of Polymorphism in Handedness

Our results suggest that strong lateralization in hand skill is an advantageous trait in men. However, natural selection has not rendered weak lateralization extinct as we still find weakly lateralized individuals in this non-industrial society. We did not have the opportunity to investigate subsequent generations and therefore we do not have the full picture concerning the fitness of our subjects. Hence, we can not be certain that eventually both strongly and weakly lateralized individuals yield the same Darwinian fitness revenues. However, the differential reproductive success found between strongly and weakly lateralized individuals reported in our study may be accurate and can possibly be maintained due to sexual antagonism. When a trait has negative effects in one sex it can persist in a population when it is advantageous in the other sex. Table 3 shows that although we found no significant associations between strength of lateralization and reproductive success in women, eight out of nine associations between strength of handedness and reproductive success are opposite in direction for men and women (binomial test: p = 0.030), suggesting possible sexual antagonism. Natural selection favouring strong lateralization in hand skill in men may thus be constrained due to possible detrimental effects on reproductive success of women.

Our results suggest that for men it may be advantageous to be strongly lateralized in hand skill, but it may be detrimental to be strongly lateralized in hand preference. Possibly, strong lateralization in hand skill may result in increased overall performance due to specialization of one of the hands. Strong lateralization in hand preference, however, may lead to reduced flexibility in hand use. This trade-off could also play a role in the persistence of different phenotypes of handedness. Although the correlation between hand preference and asymmetry in hand skill is low, it is significant and asymmetry of skill is thus to a small degree positively related to the preference to use the better performing hand. As strong hand preference may have detrimental effects this could result in a form of balancing selection possibly leading to the persistence of the different phenotypes of handedness. Furthermore, the differential selection pressures on asymmetry of hand skill and hand preference may be the underlying reason for the weak correlation between the two facets of handedness.

In order to examine whether the associations we found between strength of lateralization and reproductive success is mediated by the health of individuals we also investigated whether lateralization affected the chances of individuals of ever having experienced a severe illness or injury. We included sex as the effects of lateralization on fitness were different for men and woman. Lateralization did not in any way affect risk of severe illness; although in one case (lateralization of ball throwing) the p-value was lower than 0.1 (Table 4). However, lateralization of ball throwing was not associated with any reproductive success measurements, and its non-significant relationship with this health measure seems to be unlikely to be mediating the associations we found between strength of lateralization and reproductive success. Many studies have found a relationship between handedness and illnesses (see introduction) and the reason we did not find such an association may be explained in two ways. Individuals suffering from severe diseases may have died from these diseases, resulting in selective disappearance if handedness and health are associated (Schaafsma et al in prep.). Additionally, as no written records of health history were available and we had to rely on self-reported data, we could not reliably differentiate between different kinds of illnesses, nor evaluate their severity.

Our study shows that handedness should not be investigated solely in terms of direction, but also in terms of strength. Although this study lacks power concerning the effect of direction of hand preference due to low numbers of left-handed individuals, we did have a large data set for analyzing the effect of direction of hand skill on fitness. However, even in this case predictive factors on fitness proxies only entailed strength and not direction of handedness. Direction of lateralization follows a binary distribution (left versus right) and is therefore less informative than the continuous variable ‘strength of lateralization’. Although relationships with the latter are therefore more easily revealed, our study stresses that strength of lateralization may be just as important in explaining the evolution of handedness as is direction of lateralization.

In this study we tested handedness of individuals in which sophisticated health care and birth control is not available. Our results, although hampered by low sample size, open new research avenues for the study of the persistence of variation of handedness. Studies focussing on the differential fitness revenues of handedness between the sexes, and between hand preference and skill, will shed more light on the possibility of these specific forms of balancing selection being the mechanism underlying the different phenotypes of handedness.
